# Adverse Childhood Experiences and Disordered Eating Behaviors in Adolescence: Differences by Gender

**DOI:** 10.1007/s40653-026-00900-8

**Published:** 2026-05-09

**Authors:** Jesse J. Helton, Brandy R. Maynard, Harly M. Blumhagen

**Affiliations:** School of Social Work, Saint Louis University, St. Louis, MO USA

**Keywords:** Disordered eating behaviors, Adverse childhood experiences, Gender, Adolescents

## Abstract

Eating disorders often develop during adolescence. Disordered eating behaviors (DEBs) such as skipping meals, binging, and purging are less severe but still lead to significant health problems. Adverse childhood experiences (ACEs) are linked to various maladaptive behaviors, including DEBs, but gender-specific relationships are less understood. The purpose of this study is to explore the link between various types and measurements of ACEs and DEBs among adolescents, examining gender-specific differences. Data from the 2022 National Survey of Children’s Health (NSCH), involving a nationally representative sample of 21,573 adolescents aged 11 to 17, was used. DEBs were assessed based on parent reports of binge eating, purging, and use of diet pills. Fourteen ACEs were measured in a variety of ways, including categorized into economic hardships, violence exposure, family hardships, and discrimination. Logistic regressions modeled DEBs by ACE measures, controlling for covariates. Bivariate and multivariate analyses were stratified by gender. Each additional ACE increased odds of DEBs by 33% (OR = 1.33, *p* < .01), with no significant gender differences in cumulative effects. However, specific ACEs had gender-differentiated impacts: food insecurity increased odds among females (OR = 2.55, *p* < .01), while violence increased odds for males (OR = 3.14, *p* < .01). Body image concerns and obesity were more strongly associated with DEBs in females, whereas higher family income was significant for males. Findings highlight the need for gender-sensitive prevention and intervention strategies, addressing household stressors and promoting healthy family dynamics for females, and focusing on coping mechanisms and emotional expression for males.

Atypical eating patterns commonly develop during adolescence (Volpe et al., 2016). Eating disorders such as anorexia nervosa, bulimia nervosa, and binge eating share key characteristics: severe disruptions in eating behaviors, distressing thoughts and emotions, and significant physical, psychological, and social impairments (Vanderlinden & Palmisano, 2018). Disordered eating behaviors (DEB), though less severe, can include meal skipping, binging, purging, restrictive dieting, and the use of diet pills to control weight (Dunn et al., 2022). Globally, around 1 in 5 children and adolescents engage in DEBs (Hart et al., 2020; Lopez-Gile et al., 2023). While not everyone with DEBs will develop an eating disorder, DEBs share risk factors and outcomes with more severe disorders, including long-term consequences like health problems, self-harm, poor mental health, and impaired functioning (Kärkkäinen et al., 2018; Micali et al., 2016; Warne et al., 2021).

Both males and females engage in DEBs, but prevalence, symptoms, and impacts vary by gender (Chen et al., 2022; Massaldjieva et al., 2017). Females are more likely to restrict diets, binge eat, and purge (López-Gile et al., 2023), while DEBs in males have been increasing, especially related to body dissatisfaction, muscularity concerns, and pursuit of a lean physique (Gorrell & Murray, 2019; Mitchison & Mond, 2015). Outcomes also differ in that females may experience low body weight, amenorrhea, and osteoporosis (Barrack et al., 2021), while males are at risk of muscle dysmorphia, steroid use, and excessive exercise (Badenes-Ribera et al., 2019; Nagata et al., 2019). Gender differences in DEB etiology and consequences highlight the need for further research into how these behaviors manifest in adolescents across genders.

Various factors contribute to DEB onset and maintenance, including psychological, interpersonal, sociocultural, and biological factors. Psychological traits such as low self-esteem, negative affect, perfectionism, neuroticism, poor body image, and difficulty regulating emotions are linked to DEBs (Russon et al., 2019). These vulnerabilities often interact with environmental stressors, leading to maladaptive coping mechanisms, such as using food to manage distress. Additionally, interpersonal relationships, especially family and peer dynamics, shape attitudes toward food and body image, influencing DEB development (Haynos et al., 2016; Keel & Forney, 2013). DEBs also share risk factors with obesity, including sociocultural pressures and interpersonal dynamics (Barakat et al., 2023; Jabeile et al., 2021).

Adverse childhood experiences (ACEs) have emerged as significant risk factors for various adverse outcomes, including mental health disorders and DEBs. First studied by Felitti et al. (1998), ACEs encompass abuse, neglect, and household dysfunction, including parental incarceration, domestic violence, household mental illness or suicide, and substance abuse. Additional adversities such as neighborhood violence, bullying, and parental death are also commonly included in ACE measures. ACEs are often studied using a cumulative risk framework, with research showing a dose-response relationship: the more ACEs, the higher the likelihood of adverse outcomes (Grummitt et al., 2022; Liming et al., 2018). Some studies suggest not all ACEs are equal, with certain ACEs forming natural clusters (Lee et al., 2020), while others highlight gender differences in the association between ACEs and adverse outcomes (Grigsby et al., 2020; Jones et al., 2022; Machado et al., 2024).

ACEs are consistently linked to the development of eating disorders. Studies have shown that individuals who experience childhood adversities such as household violence, household mental illness, or neglect are more likely to develop eating disorders (Chu et al., 2022; Kovács-Tóth et al., 2022; Pop et al., 2020). The cumulative impact of ACEs also increases the likelihood of an eating disorder diagnosis (Chu et al., 2022; Guillaume et al., 2016; Kovács-Tóth et al., 2022). Specific ACEs, such as household mental illness, household violence, and criminal household members, have been found to have particularly strong associations with binge-eating disorder in younger age groups (Chu et al., 2022). Thus, childhood adversities increase the risk of developing eating disorders, and the type and number of adversities are important considerations in understanding these risks.

Fewer studies have investigated the connection between ACEs and the broader spectrum of DEBs, but some research has begun to explore these relationships. Weight-based teasing, for instance, has been linked to dietary restraint and bulimic behaviors (Menzel et al., 2010), while individual studies have identified links between physical, sexual, and emotional abuse, bullying, discrimination, and various DEBs (Dunn et al., 2022; Fuemmeler et al., 2009; Isohookanaa et al., 2016; Nelson et al., 2023). However, Morrison (2021) emphasizes the need for more research to understand how different types and numbers of ACEs relate to DEBs.

While much research has focused on the association between ACEs and eating disorders, especially in adult populations, the relationship between specific ACEs and DEBs in adolescents remains underexplored. Given that DEBs can severely impact physical and psychological well-being and increase the risk of developing clinically diagnosed eating disorders, this study aims to fill a critical gap in the literature. We will examine the relationship between multiple types of ACEs and DEBs, focusing on both individual ACE items and cumulative measures of ACEs by domain clusters. Additionally, given gender differences in DEB prevalence and emotional outcomes, the study will stratify results by gender to assess whether certain ACE items or clusters disproportionately affect adolescent females compared to males.

## Methods

Data from the 2022 National Surveys of Children’s Health (NSCH) were used for this study. The NSCH is a cross-sectional survey designed to measure child physical and mental health, as well as access to health care services and socio-environmental contexts of families. The survey employs a complex sampling methodology that draws on the US Census Bureau’s Master Address File, which spans all 50 states and the District of Columbia. NSCH data were collected using a two-stage paper survey instrument and a single-stage web-based survey instrument to ensure adequate representation across geographic regions, racial/ethnic groups, and socioeconomic status. Sample weights were calculated to account for nonresponse, align the sample distribution with the known population distribution, adjust for potential coverage error, and provide reliable estimates at the national level and for specific demographic groups. Parents or guardians responded on behalf of their children regardless of their age (CAHMI, 2024). Our sample was truncated to adolescents ages 11 to 17, resulting in *n* = 21,573.

### Measures

#### Disordered Eating

Parents were asked if their child engaged in several behaviors related to eating in the last year. These included (1) binge eating, (2) purging or vomiting after eating, or (3) using diet pills, laxative, or diuretics to lose or maintain weight without a doctor’s orders. If parents responded ‘yes’ to individual disordered eating behaviors, they were coded 1, and ‘no’ was coded as 0. If parents answered ‘yes’ to any of the three behaviors, they were coded as 1 (any disordered eating), while ‘no’ responses to any of the three items were coded as 0 (no disordered eating).

#### Adverse Childhood Experiences

Parents were asked a series of questions on household poverty, child violence exposure, family hardships, and different forms of discrimination. Each item listed below was dummy coded (yes = 1, no = 0). Four clusters of ACEs were identified: economic hardships, violence exposure, family hardships, and discrimination. *Economic Hardships* : (1) *basic needs*: reports that it was sometimes or often hard to cover basic needs like food or housing since the child was born; (2) *food scarcity*: reports that sometimes or often household was not able to afford enough food to eat in the previous year; and (3) *housing*: reports of a parent being worried about being evicted from their home in the previous year. *Violence Exposure* included: (1) the child *witnessed violence* in the home in their lifetime; (2) the child was a *victim* or witnessed violence in the neighborhood in their lifetime; and (3) the child was *bullied* or picked on by other children in the previous year. *Family Hardship* included: (1) parental *separation* or divorce; (2) parent *death*, (3) parent *incarceration*, (4) household member with severe *mental illness*, and (5) household member with *alcohol or drug problems*. Finally, *Discrimination* included: (1) *racial* discrimination: child treated or judged unfairly due to their race or ethnicity; (2) *gender* discrimination: child treated or judged unfairly due to their sexual orientation or gender identity; and (3) *disability* discrimination: child treated or judged unfairly due to health condition or disability. These 14 items were added together to create a continuous measure ranging from 0 ACEs to 14. Additionally, each of the clusters were coded as ordinal measures: 0 = no ACEs, 1 = single ACE, and 2 = multiple ACEs per cluster.

#### Covariates

Important variables related to DEBs and ACEs as identified in the literature review were included in multivariate models. These included child age in years, child sex (female or male), and race and ethnicity (Non-Hispanic Black, Hispanic, Asian, multiple race/ethnicities, Other (including American Indian or Alaskan Native, Native Hawaiian, Pacific Islander, and other race/ethnicity not listed), and Non-Hispanic White. Family poverty was measured by calculating the family’s income-to-needs ratio, which was estimated by dividing family income by its corresponding poverty threshold in 2022. The poverty threshold varies by family size and is based on the money necessary for the minimally accepted amounts of food. It ranged from 50% of the Federal poverty line (FPL) to 400% of the FPL.

Parents were asked how concerned their child was about their weight, body shape, or body size in the last year. If parents responded “very much” or “somewhat,” they were coded as 1 (*body image concern*), and “not at all” was coded as 0 (no body image concern). Parents were asked to report child’s height and weight. Body Mass Index (BMI) was then calculated as *obese* (= 1) if they were at or above the 95th percentile based on standardized growth curves published by the CDC (Kuczmarski, 2000). For family functioning, parents were asked how often all family members who live in the household ate a meal together in the previous week. Ordinal responses included never (zero days), some days (one-to-three days), most days (four-to-six days), and every day (seven days). This measure was dichotomized to *family mealtimes every day* = 1 and all else = 0.

### Analytic Plan

All analyses were performed using STATA Statistical Software Release 16. STATA survey commands were applied to obtain unbiased estimates of population parameters. The differences in DEBs by individual ACE items and ordinal cluster measures were examined using the Pearson χ^2^ test. Mean score differences in the number of total ACEs by DEB were performed using independent t-test analysis. Analyses also included logistic regressions, which modeled DEB by various measures of ACEs – including individual items, ordinal clusters, and overall mean number of items - while controlling for covariates. All analyses were also stratified by gender. Odds ratios were calculated for logistic regression models. This study was reviewed by the first author’s University institutional review board and was determined not to constitute human subjects research due to data being publicly available.

## Results

As shown in Tables 1, 6% of youth engaged in DEBs in the previous year: 0.5% purged, 5.5% binged, and 0.2% used diet pills. Regarding ACEs, 15% of families faced basic needs and housing issues, while 6% had low food security. Seven percent of children witnessed violence, 6% were victims, and 34% were bullied. 32% experienced family separation, 5% dealt with a family death, 8% had an incarcerated family member, and 12% were impacted by mental illness or substance abuse. Discrimination was reported by 7% for race, 4% for health, and 3% for gender. Youth averaged 14 years of age, with just under half being female. Racial composition was 46% non-Hispanic White, 26% Hispanic, 14% non-Hispanic Black, 9% other races, and 5% Asian. The average income-to-needs ratio was 2.5 times the poverty threshold. Body image concerns were reported by 32% of youth, with 16% classified as obese. About 33% of families ate meals together daily.

**Table 1:** Child and family characteristics (*n* = 21,573)

	%	Mean		SE	
Disordered eating					
Purging	0.5			0.01	
Binge-eating	5.5			0.03	
Pills	0.2			0.01	
Any	6			0.03	
Adversities					
Basic Needs	15			0.48	
Low Food Security	6			0.36	
Housing	15			0.52	
Witnessed	7			0.41	
Victim	6			0.35	
Bullied	34			0.63	
Separation	32			0.65	
Death	5			0.32	
Incarceration	8			0.40	
Mental Illness	12			0.45	
Substance	12			0.46	
Discrimination – Race	7			0.33	
Discrimination - Health	4			0.29	
Discrimination - Gender	3			0.18	
Child and Family Characteristics					
Child age (mean)		13.9		0.03	
Child female gender	49			0.69	
Child race and ethnicity					
Non-Hispanic Black	14			0.54	
Hispanic	26			0.73	
Asian	5			0.29	
Other	9			0.35	
Non-Hispanic White	46			0.67	
Family income-to-needs ratio		254.9		1.86	
Body image concern	32			0.63	
Obesity	16			0.56	
Family mealtimes every day	33			0.68	

All analyses were adjusted using survey weights resulting in estimates that are representative of all non-institutionalized children in the United States

T-test analysis (not reported in tables) showed that youth exhibiting a DEB had on average 3.1 ACEs (SD = 2.5) compared to 1.4 ACEs (SD = 1.7) for youth not exhibiting a DEB, t(19,593) = 30.4, *p*<.0001. There were no differences in direction, effect size, or significance when stratifying by gender. Table 2 presents results from a series of cross-tabulations with individual ACE items. Forty-one of the 42 cross-tabulations were statistically significant at the *p*<.01 level, with large effect sizes: youth who experienced any of the individual ACE items were two to four times as likely to exhibit a DEB compared to youth who did not experience the ACE item. However, female youth who experienced a parent death were not more or less likely to exhibit a DEB compared to female youth who did not experience a parent death.

**Table 2:** Adversity and disordered eating cross tabs (*n* = 21,174)

	All			Female			Male		
	%	LL	UL	%	LL	UL	%	LL	UL
Basic Needs									
Yes	12.6**	10.3	15.3	12.7**	9.8	1.6	12.4**	9.2	16.6
No	5.1	4.5	5.8	5.3	4.5	6.2	4.9	4.1	5.9
Low Food Security									
Yes	13.3**	9.7	18.0	16.3**	10.6	24.3	9.9**	6.6	14.6
No	5.8	5.2	6.5	5.8	4.9	6.6	5.8	4.8	6.9
Housing									
Yes	9.8**	7.8	12.1	10.4**	7.9	13.6	9.2**	6.4	12.9
No	5.6	4.8	6.2	5.7	4.8	6.7	5.3	4.4	6.4
Witnessed Violence									
Yes	15.3**	12.2	19.0	18.2**	13.4	24.1	12.0**	8.6	16.6
No	5.4	4.8	6.1	5.4	4.6	6.3	5.5	4.6	6.6
Violence Victim									
Yes	14.6**	11.0	19.1	13.8**	9.9	18.8	15.5**	9.9	23.4
No	5.6	5.0	6.3	5.9	5.0	6.9	5.4	4.5	6.4
Bullied									
Yes	10.8**	9.6	12.3	11.1**	9.5	13.0	10.5**	8.6	12.8
No	3.8	3.2	4.6	3.7	2.9	4.8	3.9	3.0	5.1
Parent Separation									
Yes	9.4**	8.0	11.1	9.8**	8.0	11.9	9.1**	7.0	11.7
No	4.7	4.1	5.5	4.9	4.0	6.0	4.6	3.7	5.6
Parent Death									
Yes	10.9**	7.1	16.3	8.8	5.6	13.4	12.7**	6.8	22.2
No	5.9	5.4	6.7	6.3	5.5	7.3	5.6	4.8	6.7
Parent Incarceration									
Yes	11.3**	8.9	14.2	13.6**	10.1	18.0	9.3*	6.2	13.7
No	5.7	5.1	6.4	5.7	4.9	7.0	5.7	4.8	6.8
Mental Illness									
Yes	16.0**	13.4	19.0	16.7**	13.2	20.1	15.1**	11.4	19.8
No	5.0	4.3	5.7	4.9	4.1	5.8	5.0	4.1	6.1
Substance Problem									
Yes	14.0**	11.6	16.9	15.6**	12.2	19.9	12.2**	9.0	16.3
No	5.1	4.5	5.9	5.1	4.3	6.1	5.2	4.3	6.3
Discrimination – Race									
Yes	10.1**	7.9	14.7	12.0**	8.8	16.3	9.5**	5.0	17.1
No	5.9	5.2	6.6	5.9	5.1	6.9	5.8	4.9	6.8
Discrimination - Disability									
Yes	20.1**	16.5	25.2	19.6**	13.8	21.2	21.3**	16.2	27.4
No	5.5	4.9	6.2	5.8	5.0	6.8	5.3	4.4	6.4
Discrimination - Gender									
Yes	16.0**	11.8	21.2	17.4**	12.7	23.5	12.4**	5.9	25.4
No	5.9	5.2	6.6	5.9	5.1	6.9	5.8	5.0	6.8

**p<.01 *p<.05

*LL* Lower level of 95% confidence interval, *UL* Upper level of 95% confidence interval, All analyses were adjusted using survey weights resulting in estimates that are representative of all non-institutionalized children in the United States.

**Fig. 1:**
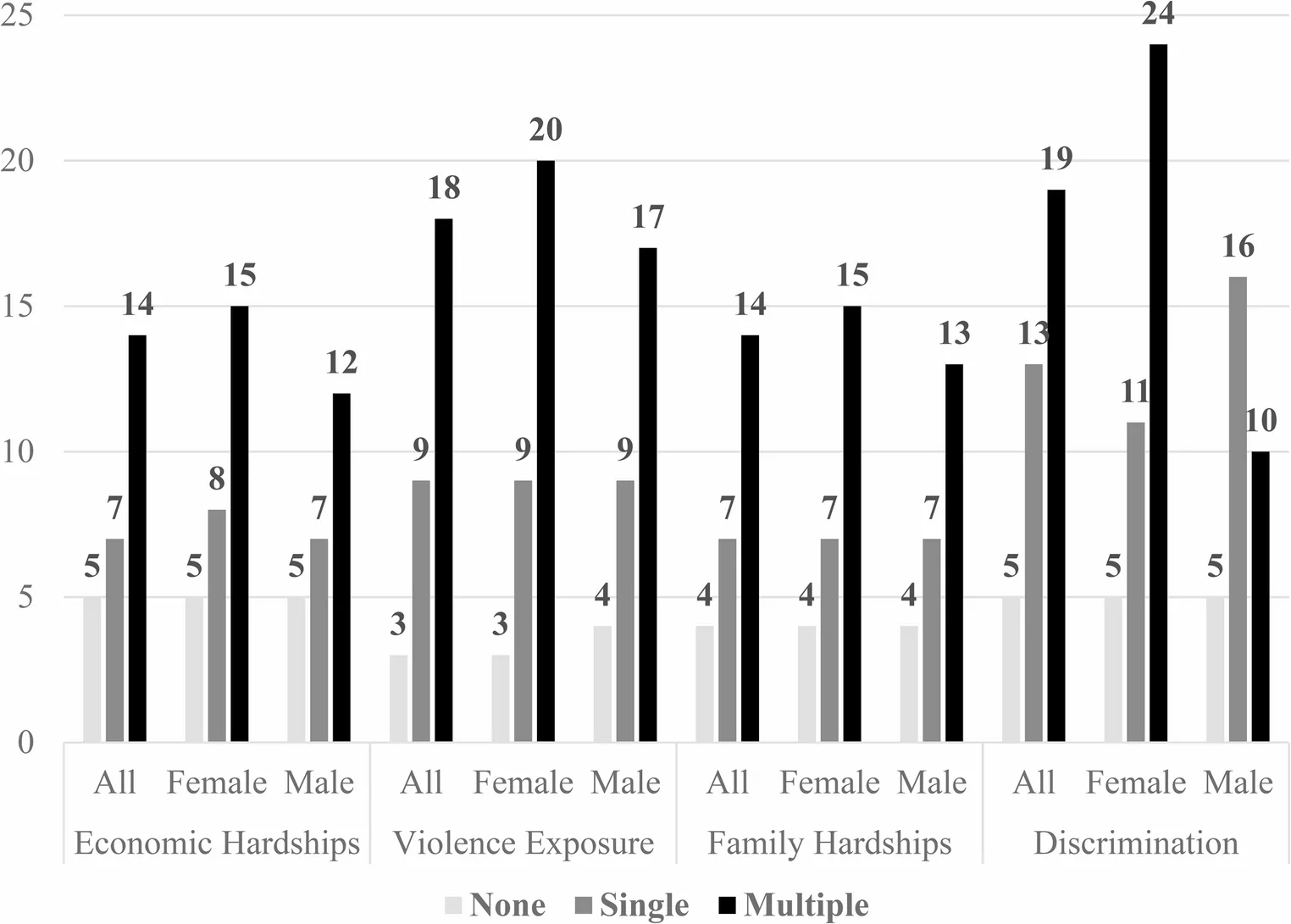
Percentage of adolescent youth exhibiting a disordered eating behavior by type and number of adverse childhood experience clusters stratified by gender

Figure 1 reports the results of multiple cross-tabulations of the ordinal measure of ACE exposures in different clusters stratified by gender. Overall, rates of DEB increased in lock-step with the number of different ACE exposures within clusters, and the direction, effect size, and statistical significance did not change based on gender, with one exception. While 24% of female youth who experienced multiple forms of discrimination exhibited a DEB compared to 11% who experienced a single form and 5% who did not experience a discrimination ACE, χ^2^(2, *n* = 9,886) = 32.4, *p*<.001, only 10% of male youth who experienced multiple forms of discrimination exhibited a DEB compared to 16% who experienced a single form of discrimination and 5% who did not experience discrimination, χ^2^(2, *n* = 10,569) = 33.4, *p*<.001.

Table 3 presents the results of three logistic regressions modeling a DEB by cumulative measures of all adversities, with controls, and stratified by gender. For both genders, each additional ACE was associated with a 33% increase in the odds of engaging in a DEB (OR = 1.33, *p*<.01). This association was similarly significant for both females (OR = 1.29, *p*<.01) and males (OR = 1.38, *p*<.01). Body image concern was associated with increased odds of a DEB, with a stronger association observed among females (OR = 2.75, *p*<.01) compared to males (OR = 1.97, *p*<.01). Obesity also substantially increased the odds of disordered eating, with females showing slightly higher odds (OR = 3.45, *p*<.01) compared to males (OR = 2.99, *p*<.01). Daily family mealtimes decreased odds for DEBs for females (OR = 0.65, *p*<.05), but this effect was not observed in males. Additionally, family income was a significant predictor for males, with higher incomes associated with greater odds of a DEB (OR = 1.02, *p*<.05); this was not observed for females.

**Table 3:** Logistic regression predicting disordered eating by cumulative measure of all adversities with controls, stratified by gender

	All			Female			Male		
	OR	LL	UL	OR	LL	UL	OR	LL	UL
ACEs	1.33**	1.27	1.40	1.29**	1.21	1.38	1.38**	1.29	1.47
Child age (mean)	1.01	0.95	1.07	1.02	0.93	1.11	1.00	0.93	1.09
Child female gender	1.09	0.83	1.44						
Child race and ethnicity									
Non-Hispanic Black	0.87	0.51	1.50	0.61	0.31	1.23	1.16	0.55	2.46
Hispanic	1.12	0.78	1.60	1.21	0.77	1.87	1.04	0.57	1.89
Asian	1.02	0.58	1.80	1.30	0.64	2.64	0.83	0.33	1.92
Other	1.33	0.92	1.92	1.61	0.94	2.76	1.04	0.63	1.68
Family income	1.00	0.99	1.01	0.99	0.98	1.01	1.02*	1.01	1.03
Mealtimes every day	0.85	0.63	1.17	0.65*	0.43	0.99	1.11	0.71	1.73
Body image concern	2.32**	1.75	3.06	2.75**	1.88	3.99	1.97**	1.31	2.99
Obese	3.13**	2.36	4.13	3.45**	2.34	5.09	2.99**	2.06	4.34

**p<.01 *p<.05

Table 4 presents the results of 54 logistic regressions, each modeling a DEB by individual ACE items as well as ordinal ACE clusters, with controls, and stratified by gender. Overall, youth experiencing basic needs insecurity were more than twice as likely to engage in a DEB (OR = 2.15, *p*<.01). Low food security significantly increased the odds of a DEB in the overall sample (OR = 1.91, *p*<.01) and for females specifically (OR = 2.55, *p*<.01), but not for males. Housing insecurity was a significant predictor in the overall sample (OR = 1.49, *p*<.05), but not when stratified by gender. Experiencing multiple economic adversities significantly increased the odds of a DEB in the overall sample (OR = 2.36, *p*<.01). Being a victim of violence had a notable impact on males (OR = 3.14, *p*<.01) but not females. Exposure to bullying was a significant predictor for both genders (OR = 2.88, *p*<.01). Experiencing multiple forms of violence exposure dramatically increased the odds of a DEB in the overall sample (OR = 4.91, *p*<.01).

**Table 4:** Logistic regression predicting disordered eating by individual ACE items, ordinal ACE clusters, with controls, stratified by gender

	All			Female			Male		
	OR	LL	UL	OR	LL	UL	OR	LL	UL
Basic Needs	2.15**	1.55	2.97	1.72**	1.16	2.54	2.51**	1.56	4.07
Low Food Security	1.91**	1.25	2.91	2.55**	1.41	4.59	1.27	0.72	2.26
Housing	1.49*	1.07	2.09	1.39	0.91	2.13	1.56	0.94	2.61
Economic Hardship cluster									
None	ref			ref			ref		
Single	1.26	0.91	1.78	1.04	0.65	1.66	1.45	0.89	2.35
Multiple	2.36**	1.55	3.59	2.17**	1.31	3.60	2.39**	1.25	4.57
Witnessed	2.36**	1.73	3.21	2.58**	1.66	4.01	2.04**	1.32	3.16
Victim	2.25**	1.52	3.34	1.51	0.96	2.35	3.14**	1.77	5.56
Bullied	2.88**	2.21	3.78	2.83**	1.98	4.04	2.89**	1.94	4.32
Violence Exposure cluster ACE									
None	ref			ref					
Single	2.48**	1.83	3.34	2.49**	1.67	3.73	2.41**	1.55	3.74
Multiple	4.91**	3.39	7.11	4.66**	2.92	7.40	4.98**	2.81	8.78
Separation	1.75**	1.13	2.28	1.67**	1.15	2.41	1.87**	1.27	2.76
Death	1.74	0.97	3.09	1.36	0.81	2.29	2.00	0.83	4.86
Incarceration	1.64**	1.15	2.31	1.65*	1.02	2.64	1.55	0.92	2.63
Mental Illness	2.82**	2.15	3.69	2.51**	1.78	3.55	3.09**	2.06	4.61
Substance	2.31**	1.76	3.04	2.28**	1.56	3.12	2.37**	1.56	3.63
Family Hardship cluster ACE									
None	ref			ref					
Single	1.78**	1.25	2.53	1.54	0.95	2.52	2.08**	1.27	3.40
Multiple	3.13**	2.33	4.21	2.61**	1.74	3.89	3.68**	2.41	5.61
Discr – Race	1.52	0.99	2.32	1.59	0.99	2.65	1.40	0.67	2.92
Discr - Health	3.66**	2.69	4.95	2.52**	1.64	3.88	4.72**	3.12	7.13
Discr - Gender	2.01**	1.32	3.04	2.02**	1.27	3.21	1.72	0.65	4.56
Discrimination Cluster ACE									
None	ref			ref			ref		
Single	2.37**	1.74	3.22	1.46	0.97	2.18	3.52**	2.34	5.29
Multiple	2.76**	1.82	4.18	3.07**	1.81	5.20	1.81	0.89	3.67

**p<.01 *p<.05

All analyses controlled for child age, race gender (when not stratified), family income, family mealtimes, body image concerns, and obesity.

Parent separation also significantly increased the odds of a DEB (OR = 1.75, *p*<.01). Parental death was not associated with DEBs. Incarceration was a predictor across all groups (OR = 1.64, *p*<.01), but was only associated with DEB for females (OR = 1.65, *p*<.05) and not males. Other significant predictors included mental illness in the family, which raised the odds for both females (OR = 2.51, *p*<.01) and males (OR = 3.09, *p*<.01), and substance abuse in the family (OR = 2.31, *p*<.01), which also elevated the odds for both genders. Experiencing multiple forms of family hardships compared to none increased the odds of a DEB in the overall sample (OR = 2.36, *p*<.01). Experiencing discrimination based on race was not associated with DEBs, but experiencing discrimination based on disability had a substantial impact on both genders, with the effect being particularly strong for males (OR = 4.72, *p*<.01). Gender-based discrimination significantly increased the odds for females (OR = 2.02, *p*<.01) but not for males. The cumulative measure of experiencing multiple forms of discrimination significantly raised the odds of DEBs for females (OR = 3.07, *p*<.01) but not for males, whereas experiencing a single form of discrimination significantly raised the odds of DEB for males (OR = 3.52, *p*<.01) but not for females.

## Discussion

Our study began with the hypothesis that gender played an important role in the relationships between specific types of ACEs - not simply cumulative scores - and DEBs. Logistic regressions revealed that a cumulative measure of ACEs heightened DEB likelihood by 33% overall, which was consistent with previous findings (Chu et al., 2022). Overall, even when controlling for important covariates, twelve out of fourteen ACE types were associated with DEBs, and eight out of fourteen were associated with DEBs across both genders. Experiencing multiple forms of violence was associated with the highest risk of DEB. However, there were also important differences in DEB association with certain individual ACE items based on gender. Most notably, food insecurity heightened DEB risk among females and exposure to violence heightened DEB risk for males. Further, body image concerns and obesity were more strongly associated with DEBs in females, while eating frequent meals together lowered the odds of DEBs for females. These findings underscore the importance of gender-specific approaches in ACE research, informing targeted interventions to mitigate DEBs and promote adolescent health.

It is difficult to theoretically unpack why female adolescents’ DEB risk was associated with mostly household-level factors - food insecurity, parent incarceration, and family mealtime frequency - while risk for males was associated with violence and family income. Our measure of DEB was mostly binge eating behaviors. Binge eating is typically characterized by eating large quantities of food in a short period of time. Binging has been reported by adults with eating disorders as a way to control negative emotions (Cooper et al., 2004) and reduce anxiety and stress (Pallister & Waller, 2008). Because of the connection to family-level stresses, family system theory may be a worthwhile theoretical start. Family Systems theory posits that girls are more likely to take on caregiver roles and be more attuned to the emotional needs of the family unit (Hughes et al., 2008). Food insecurity, which is measured in our study as a household adversity, may exacerbate these gender roles, leading girls to experience higher stress about their family’s well-being. 

Understanding why males may be at higher risk than females for developing DEBs in response to violence could involve several factors. Cultural norms and societal expectations often discourage males from showing vulnerability, leading to emotional suppression (Kerig, 1999). Male adolescents in higher-income families may also feel pressured to suppress emotions due to expectations of success and self-reliance (Plant et al., 2000). This suppression may cause DEBs to emerge as a coping mechanism, using food to express emotions or regain control. Additionally, research suggests females are more likely to seek help and have stronger social connections that buffer the effects of violence (Eschenbeck et al., 2007).

Several limitations of the current study warrant consideration. First, the cross-sectional design precludes establishing causality. Second, reliance on parental reporting may introduce bias, particularly in sensitive areas like DEBs and ACEs. Parents may underreport behaviors or experiences due to social desirability bias or lack of awareness. Third, our measures of DEBs are limited to three behaviors, potentially omitting other relevant disordered eating patterns. Fourth, while the sample is large and nationally representative, the findings may not generalize to all subpopulations, especially those underrepresented in the survey. For instance, we were not able to stratify by race, sexual orientation, or disability status when examining discrimination’s association with DEBs.

Given the observed gender differences found in the associations between individual ACEs and DEBs, adopting a gender-sensitive approach to prevention and treatment may be beneficial. A one-size-fits-all approach may not be effective, as males and females are impacted by and respond to trauma and adverse experiences differently which may generate different pathways for males and females to develop DEBs. Moreover, research suggests that men are less likely than women to see the need for treatment and less likely to seek help for disordered eating for a variety of reasons including stigma and barriers to treatment due to the misperceptions that disordered eating is a female problem and masculine gender socialization that prioritizes self-reliance (Bunnell, 2021). Taking a gender-based approach to prevention and treatment strategies can address the unique psychological, social, and biological needs of each gender as well as social and cultural barriers to treatment. The critical significance of early identification and support for adolescents exposed to ACEs in mitigating the risk of developing DEBs cannot be overstated.
